# Pre-clinical development of the human CD40 agonistic antibody ADC-1013

**DOI:** 10.1186/2051-1426-3-S2-P185

**Published:** 2015-11-04

**Authors:** Per Norlén, Niina Veitonmäki, Karin Enell Smith, Thomas H Tötterman, Sara M Mangsbo, Christina Furebring, Peter Ellmark

**Affiliations:** 1 Alligator Bioscience AB, Lund, Sweden; 2 Immunology, Genetics and Pathology, IGP, Uppsala University, Uppsala, Sweden

## 

Activation of CD40 on dendritic cells increases cross-presentation of tumor antigens and as a result the number of activated tumor directed T effector cells. CD40 agonists have been shown to generate powerful systemic anti-tumor responses when administered in situ.

The pre-clinical proof of concept data has been generated mainly using an hCD40 transgenic mouse (hCD40tg) strain. Anti-tumor effects has been demonstrated in multiple syngeneic tumor models including bladder tumor (MB49), melanoma (B16) and lymphoma (A20) models. The anti-tumor effects has been shown to induce long term, T cell dependent, tumor immunity. As expected, the anti-tumor effects depends on the immune status of the tumors as evaluated by immunohistochemistry and flow cytometry.

Since CD40 agonistic antibodies mainly exert their effects upstream of the checkpoint inhibitors they have the potential to be ideal candidates for combination regimens including e.g. PD-1 antagonists and OX40 antibodies. Combining ADC-1013 with antibodies against these targets has been shown to improve the anti-tumor effects in B16.F10 melanoma.

Toxicology studies were performed in cynomolgus monkeys using subcutaneous or intravenous administration. Doses of 1 to 10 mg/kg were evaluated in both studies. ADC-1013 was well tolerated at all dose levels and no major safety concerns were identified. Receptor saturation of approximately 95% was achieved and pharmacodynamic activity, notably B-cell depletion, was observed at all dose levels.

In cynomolgus monkey, serum concentrations of ADC-1013 declined in a multi-phasic manner following a single intravenous dose. The mean terminal half-life was approximately 30 hours. Following subcutaneous administration the mean elimination half-life was found to be between 0.6 and 3.2 days. The clearance (CL_F) decreased and the half-life increased with increasing dose indicating target-mediated clearance.

Alligator Bioscience has recently started dosing of the first patients in a clinical trial of the CD40 agonistic antibody ADC-1013. The study is a first-in-human, multicenter, open-label, multiple ascending dose Phase I study in patients with advanced solid tumors to determine the safety, pharmacokinetics and pharmacodynamics of intratumorally administered ADC-1013.

